# Dihydromyricetin Inhibits Tumor Growth and Epithelial-Mesenchymal Transition through regulating miR-455-3p in Cholangiocarcinoma

**DOI:** 10.7150/jca.61311

**Published:** 2021-08-22

**Authors:** Xin Li, Zhou-Sheng Yang, Wen-Wu Cai, Yang Deng, Lei Chen, Sheng-Lan Tan

**Affiliations:** 1 Department of Vascular Surgery, the Second Xiangya Hospital, Central South University, Changsha, Hunan, China, 410011.; 2 The Institute of Vascular Diseases, Central South University, Changsha, Hunan, China, 410011.; 3 Department of Pharmacy, The People's Hospital of Guangxi Zhuang Autonomous Region, Nanning, Guangxi, China, 530021.; 4 Department of General Surgery, The Second Xiangya Hospital, Central South University, Changsha, China, 410011.; 5 Department of pharmacy, The Third Hospital of Changsha, Changsha, China, 410015.; 6 Department of Pharmacy, The Second Xiangya Hospital, Central South University, Changsha, China, 410011.; 7 Institute of Clinical Pharmacy, Central South University, Changsha, China, 410011.

**Keywords:** Dihydromyricetin, cholangiocarcinoma, miR-455-3p, epithelial-mesenchymal transition

## Abstract

Cholangiocarcinoma (CCA) leads to poor prognosis due to high aggressiveness and common chemoresistance. Dihydromyricetin (DMY), the main bioactive compound isolated from Ampelopsis grossedentata, exhibits broad anti-tumor effects. This study aimed to investigate the inhibitory effect of DMY on CCA tumor growth and epithelial-mesenchymal transition (EMT) and its underlying mechanism in CCA. DMY treatment significantly inhibited cell proliferation and EMT in CCA cell lines. The expression of ZEB1 and vimentin were down-regulated, while the level of E-cadherin was increased after DMY treatment. By analyzing the TCGA dataset, we found that miR-455 expression was significantly downregulated, while the level of ZEB1 was up-regulated in human CCA tumor tissues compared to normal samples. Mechanistic studies showed that ZEB1 was a direct target of miR-455-3p in CCA. Moreover, DMY treatment potently increased miR-455-3p expression and inhibited ZEB1 expression. Inhibition of miR-455-3p expression abolished DMY's inhibitory effects on tumor growth and EMT in both CCA cells and cell-engrafted nude mice. Finally, DMY significantly suppressed the expressions of p-PI3K and p-AKT, while silencing miR-455-3p remarkably abrogated the inhibitory effect. In conclusion, DMY suppresses tumor growth and EMT through regulating miR-455-3p in human cholangiocarcinoma, suggesting a potential option for CCA treatment.

## Introduction

Constituting approximately 15% of all primary liver tumors, cholangiocarcinoma (CCA) is the second most common primary hepatic malignancy after carcinoma 1. According to their anatomical locations, CCAs are divided into three categories as intrahepatic (iCCA), perihilar (pCCA), and distal (dCCA). The incidence and mortality of CCA are increasing globally in the past decades, placing a worldwide health problem 2. The prognosis of CCA patients is still disappointing, with low 5-year survival (7-20%) 2. Surgery is the best option for CCA, however, up to 70% patients are diagnosed at late stages due to no specific symptoms, resulting in no chance of surgery and can only receive palliative treatment 2, 3. The cisplatin plus gemcitabine chemotherapy is the first-line regimen for patients with advanced CCA. Besides a lot of side effects, the major limitation of chemotherapy in the management of patients with advanced CCA is the marked multi-drug resistance phenotype of CCA 2. Therefore, studies of new therapeutic agents for CCA treatment are urgently required to improve patient medical outcomes.

Due to the unsatisfactory effect of traditional cytotoxic drugs in the treatment of CCA, it is promising to develop new drugs based on other mechanisms of tumor pathogenesis. Epithelial to mesenchymal transition (EMT) is defined as a cell biological program, during which epithelial cells progressively lose their junctions and apical-basal polarity, and gradually obtain mesenchymal characteristics 4. EMT is a relatively reversible process and mesenchymal-epithelial transition (MET) describes the reverse procedure. Accumulating evidences demonstrate that EMT not only plays an important role in embryogenesis, organ development, tissue regeneration and organ fibrosis, but also contributes greatly to cancer development and progression 5, 6. For instance, cancer cells promote their ability of migration, invasion and metastasis via the mechanism of EMT. Moreover, EMT is also involved in reducing sensitivity of cancer cells to chemotherapy. During EMT, cancer cells alter a series of gene expressions, including downregulation of epithelial markers (e.g. E-cadherin) and upregulation of mesenchymal markers (e.g. N-cadherin and vimentin) 4.

Natural plant compounds have been used for decades as adjuvant therapy and a structural basis for drug development. Many bioactive compounds isolated from natural plants can regulate EMT through the mechanisms of anti-inflammation, anti-fibrosis or antioxidation 7. Dihydromyricetin (DMY), a natural flavonoid and the main active compound isolated from the plant Ampelopsis grossedentata, has broad spectrum and multiple target effects in several cancers with low toxicity, suggesting a high and promising possibility of DMY in cancer treatment 8-10. We previously found DMY significantly inhibited CCA cell migration and invasion via inhibiting miR-21 expression 11. Yet, overexpression of miR-21 only partly abrogated the DMY's anti-tumor effects in CCA, which promotes us to discover a comprehensive mechanism of how DMY exerts its anti-tumor effects.

MicroRNAs (miRNAs) are single stranded non-coding RNAs with a length of 19 to 24 nucleotides that negatively regulate the expression of target genes. Studies have demonstrated that multiple miRNAs are involved in cancer metastasis and progression through controlling EMT processes including in CCA 12. For instance, a previous study showed that miR-186 inhibited CCA cell proliferation and EMT through targeting Twist1 13. MiR-455 has been shown to be down-regulated in several gastrointestinal cancers, including gastric cancer and pancreatic cancer 14, 15. However, the role of miR-455 in CCA is still largely unknown. By analyzing the TCGA dataset, we identified that the expression of miR-455 in human CCA tissues was also significantly decreased compared to the non-tumor tissues. As the previous study showed that miR-455 exerted anti-cancer effects in non-small cell lung cancer through targeting the EMT inducible transcription factor ZEB1 16, we aimed to investigate if the miR-455/ZEB1 axis was also involved in EMT in CCA in this study.

In the current study, we investigated the effects of DMY on tumor growth and EMT in human CCA both *in vitro* and *in vivo*. For the first time, we found that DMY significantly inhibited tumor growth and EMT by targeting the miR-455/ZEB1 axis, suggesting DMY might be explored as a potential candidate for the treatment of CCA.

## Material and Methods

### Cell culture and treatment

Human CCA cell lines including RBE and TFK-1 were purchased from American Type Culture Collection (Rockville, MD, USA). The cells were maintained in RPMI-1640 medium (Gibco, Thermo Fisher Scientific, MA, USA) containing 10% fetal bovine serum (Gibco) in a humidified atmosphere at 37 °C with 5% CO_2_. Different concentrations of dihydromyricetin (Sigma-Aldrich, St Louis, MO, USA) dissolved in dimethyl sulfoxide (DMSO, Sigma-Aldrich) were tested and 150 μM was finally selected for treatment.

### Cell viability assay

The Cell Counting Kit-8 (CCK-8) (Dojindo, Japan) assay was used to assess cell viability after exposure to different concentrations of dihydromyricetin. Briefly, RBE cells were seeded in 96-well plates at a density of 5×10^4^ cells per well and were treated with different concentrations of dihydromyricetin (10, 50, 100, 150, 200 and 400 μM) for 24 hours. After treatment, cell culture medium was replaced with fresh medium containing10 μL CCK-8 solution in each well, and cells were incubated for 1 h at 37 °C with 5% CO2. The absorbance of the solution was measured at 450 nm using a Microplate Reader (Bio-Rad, CA, USA).

### Colony formation assay

Suspensions of single RBE cells were prepared and seeded in six-well plates. Cells were maintained in RPMI-1640 supplemented with 10% fetal bovine serum containing different treatment solution at 37°C and 5% CO2 for about 2 weeks. When single-cell clones become visible, the colonies were washed with PBS twice, followed by fixed in 4% paraformaldehyde for 15 min. Then the colonies were stained with crystal violet (Beyotime, Beijing, China). Finally, colonies were counted under a microscope (Olympus Corp., Tokyo, Japan).

### Cell proliferation assay

The EdU assay (Beyotime, Beijing, China) was used to measure cell proliferation according to the manufacturer's instructions. Cells were plated in 96-well plates at a density of 5×10^4^ cells per well. After treatments, cell culture medium was replaced with pre-warmed fresh medium containing 10 μM EdU, and cells were incubated for 3 hrs. Then cells were fixed in 4% Paraformaldehyde for 15 min and incubated in 0.3% Triton-X 100 for 15 min at room temperature. After washed with PBS for three times, cells were incubated with the prepared Click buffer for 30 min in dark at 37 °C, followed by counterstained with Hoechst for 10 min. Finally, EdU-positive cells, DAPI-labeled cells and their merged images were captured under a fluorescence microscope (Zeiss, Jena, Germany).

### Cell invasion and migration assays

Transwell chambers with 8 μm size of pores (BD Biosciences, CA, USA) were used to assess the migration and invasion of cells same as our previous study 11. For cell invasion evaluation, chambers were coated on the upper side with BD MatrigelTM Matrix (BD). After treatment, 8×10^4^ cells suspended in 200μl of serum free medium were added to the upper chamber, and the lower chambers were supplemented with medium containing 10% FBS. After incubated for 24 hrs, cells remaining in the upper chamber were completely removed by gently swabbing. Cells which migrated to the lower chamber were fixed with 4% paraformaldehyde for 15 min and stained with 0.4% crystal violet (Merck, Germany) for 15 min. Finally, cells were counted and photographed under microscope. The method of cell migration was similar, except using chambers without BD Matrigel.

### RNA isolation and quantitative real-time polymerase chain reaction

RNA level was measured by RT-qPCR. Total RNA was extracted from tumor tissues and cells by TRIzol regent (Invitrogen, CA, USA) and then reversely transcribed into cDNA. The commercial Reverse Transcribed Kit (Takara, Dalian, China) was used to synthesize cDNA, and real-time qPCR was conducted using the TB Green real-time PCR kit (Takara, Dalian, China). U6 was used as an internal control. The primer sequences used were: ZEB1 forward, 5′-GCACCTGAAGAGGACCAGAG-3′; ZEB1 reverse, 5′-GTGTAACTGCACAGGGAGCA-3′. glyceraldehyde-3-phosphate dehydrogenase (GAPDH) forward, 5′-CTGCACCACCAACTGCTTAG-3′, reverse, 5′-AGGTCCACCACTGACACGTT-3′. The primers for miR-455-3p (MQPS0001464-1-100) and U6 (MQPS0000002-1-100) were ordered from RiboBio (Guangzhou, China).

### Screening of differentially expressed genes in human CCA

The RNA-seq and miRNA-seq data of cholangiocarcinoma were downloaded from the TCGA database. The R software was used to standardize and log2 transform the data. TCGA ID was used to match the expression of ZEB1 and miR-455. Spearman correlation coefficient was calculated and statistically tested. The results were displayed with R package “ggpubr”.

### Cell transfection

RBE cells were transfected with miRNA non-specific mimic control (NS-m) (miR1N0000001-1-5, Ribobio, Guangzhou, China), miR-455-3p mimic (455-m) (miR10004784-1-5, Ribobio, Guangzhou, China), miRNA non-specific inhibitor (NS-i) (miR2N0000001-1-5, Ribobio, Guangzhou, China), miR-455-3p inhibitor (455-i) (miR20004784-1-5, Ribobio, Guangzhou, China) at a final concentration of 100 nM following the manufacturer's protocol. Cells were transiently transfected by use of Lipofectamine 2000 transfection reagent from Invitrogen (11668019, USA). After 14 to16 hrs of incubation, fresh culture medium was replaced and cells were incubated for another 12 hours before harvested for protein or RNA analyses. Real time qPCR was used to confirm that miR‑455 expression was specifically up-regulated or down-regulated after transfection.

### Western blot assay

Tumor tissues were first homogenized using TissueLyser II (Qiagen, USA) according to manufacturer's instructions. Then proteins from homogenized tumor tissues and CCA cell lines were lysed with the radio immunoprecipitation assay (Beyotime, Beijing, China). Proteins were separated by sodium dodecyl sulfate-polyacrylamide gel electrophoresis (SDS-PAGE) using a 10% gradient gel and transferred onto 0.45 μm polyvinylidene fluoride (PVDF) membranes (Millipore, Billerica, MA, USA). After blocking nonspecific binding with TBS-T (0.1% tween) containing 5% non-fat milk for 1h at room temperature, the membranes were incubated with primary antibodies at 4 °C overnight and then secondary antibodies for 1 h at room temperature. The protein bands were detected using electrochemiluminescence (ECL) (Beyotime, Beijing, China), and band quantification was analyzed using the Image Lab™ Software (Bio-Rad, USA). The primary antibodies used in this study were as follows: ZEB1 (1:1000, 21544-1-AP, Proteintech, China), E-cadherin (1:5000, 20874-1-AP, Proteintech, China), vimentin (1:1000, 3932, CST, USA), phospho-PI3K (1:1000, AP0854, Abclonal, China), phospho-Akt (1:1000, 4060, CST, USA), and GAPDH (1:1000, ab8245, Abcam, UK).

### Dual-luciferase reporter assay

The wild-type (WT) and mutant-type (Mut) 3'-untranslated regions (UTRs) of ZEB1 were synthesized and cloned to pmirGLO Dual-Luciferase miRNA Target expression vector (E1330, Promega, USA) by Vigene Biosciences (Shandong, China) according to the manufacturer's instructions. HEK 239T cells (1.5 × 10^5^ cells per well) were seeded on 12-well plates and co-transfected with 250 ng WT or Mut luciferase reporter constructs, 10 ng Renilla plasmid (E2231, Promega, USA), 100 nM 455-m or NS-m, together with lipofectamine 2000 after cells growing to 70% confluency. The cell lysates were collected 24 hours after transfection, and the luciferase activities were measured using the Dual-Glo Luciferase Assay System (E2920, Promega, USA). A fluorescence reader (Veritas 9100-002, Turner Biosystems, USA) was used and each reading of luciferase activity was normalized to the Renilla activity.

### Nude mice xenograft tumorigenesis studies

All animal experiments were approved by the Ethics Committee for Laboratory Animals of The Second Xiangya Hospital of Central South University, Hunan, China, and followed the Interdisciplinary Principles and Guidelines for the Use of Animals in Research, Testing, and Education by the New York Academy of Sciences, Ad Hoc Animal Research Committee. RBE cells (5×10^6^) were resuspended in 50μL RPMI-1640 medium mixed with 50 uL Matrigel. Then cells were subcutaneously injected into the right flank of male four-week-old Balb/c nude mice (Hunan SJA Laboratory Animal Cooperation, Changsha, Hunan, China). Three days after injection, mice were randomly divided into two groups (n = 6 mice per group) and intragastric treated with DMY (500 mg/kg/day) for 4 weeks. Intratumoral injection with lipofectamine 2000 encapsulated 455-i or NS-i (5 nM/mouse) was according to protocol previously described 17. Animals were maintained under specific pathogen-free conditions, and animal protocols were reviewed and approved by the Ethics Committee for Laboratory Animals of The Second Xiangya Hospital, Central South University, Hunan, China. All mice were euthanized 4 weeks after injection, and the xenograft tissues were harvested and fixed with 10% formalin solution for immunohistology analysis.

### Immunohistochemistry

The tissue paraffin sections were heated in an oven at 60 °C for 2 h, followed by dewaxed with xylene for 20 min, and dehydrated with gradient ethanol solution. Then sections were washed with PBS three times, incubated in 3% hydrogen peroxide for 10 min, washed with PBS three times again, and put in sodium citrate buffer (10 nM sodium and 0.05% Tween 20 at pH 6.0) at 96 °C for 30 minutes for antigen repair. After being blocked with calf serum for 20 min, primary antibodies were added and incubated for 60 minutes at room temperature, including Ki67 (1: 50, A2094, Abclonal), ZEB1 (1:50, 21544-1-AP, Proteintech, China), E-cadherin (1:500, 20874-1-AP, Proteintech, China) and vimentin (1:1000, 3932, CST, USA). After rinsed with PBS three times, the secondary antibodies were incubated at 37 ° C for 30 min, washed with PBS three times, and developed with DAB (ZLI-9017, Zsbio, China). Sections were then counterstained with hematoxylin, dehydrated with ethanol, cleared with xylene, and mounted. All images were captured using a Microscope VS120 Whole Slide Scanner (Olympus) and analyzed using the computer-assisted Image-Pro Plus software (Meida Cybernetics, Bethesda, MD). The quantifications of Ki67, and ZEB1, E-cadherin and vimentin staining were performed as positive area percent. All specimens were confirmed by two independent observers.

### Statistical analysis

All data were analyzed using the SPSS 22.0 statistical software (IBM Corp., NY, USA) and GraphPad Prism 5.01 (GraphPad Software, CA, USA) and data were presented as mean ± standard deviation. Each experiment was performed in triplicates for at least three times. Comparisons between two groups or multiple groups were performed using the independent t-test or one‑way analysis of variance (ANOVA). Spearman correlation was used to calculate the coefficient between miR-455 and ZEB1. * *P* < 0.05, ** *P* < 0.01 and *** *P* <0.001 were considered statistically significant.

## Results

### DMY inhibits cell growth and EMT in CCA cell lines

**Figure 1:**
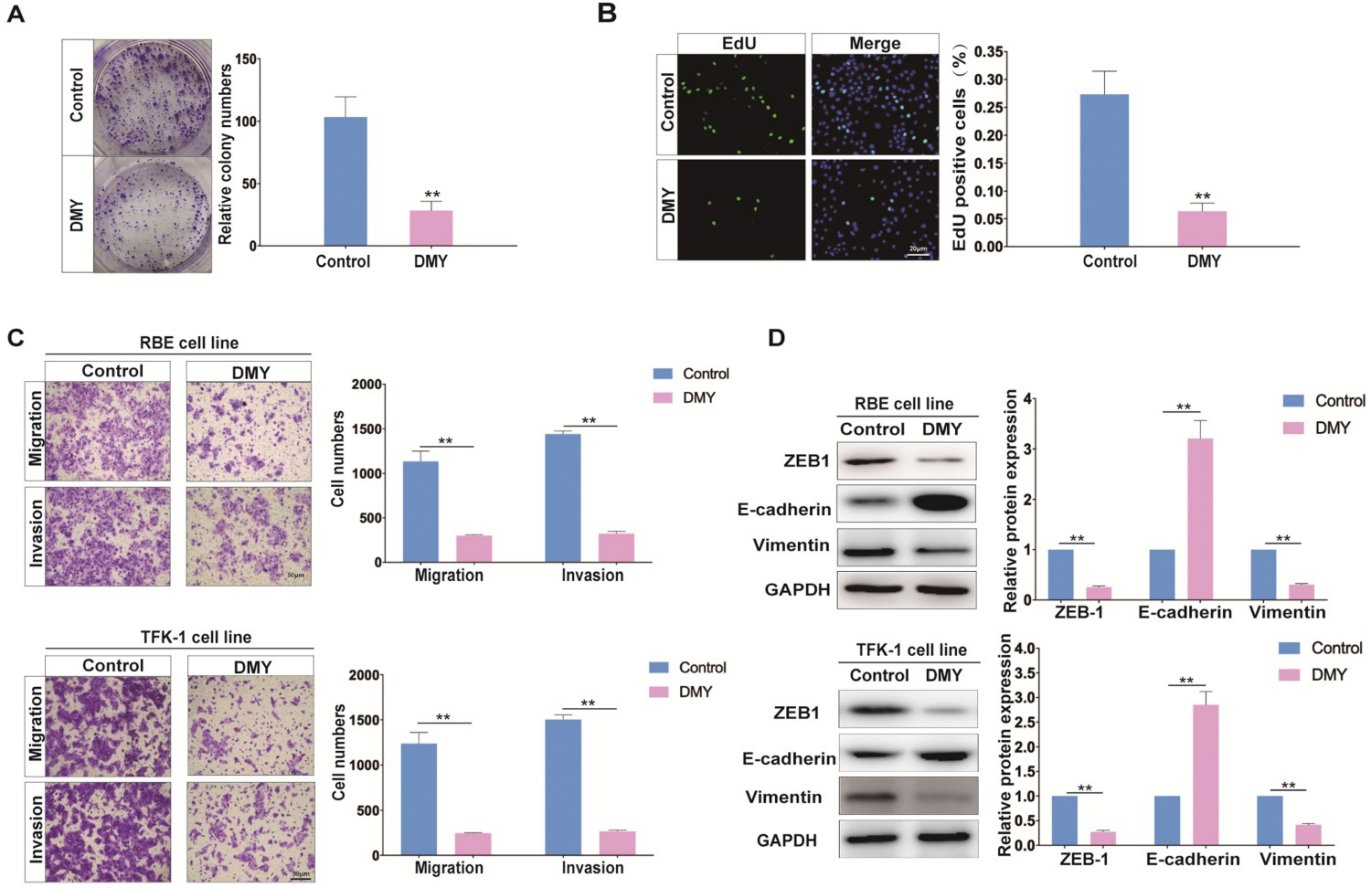
Dihydromyricetin inhibits cell growth and epithelial to mesenchymal transition (EMT) in RBE cells and TFK-1 cells. (**A**-**B**) Colony formation assay and EdU assay were used to determine cell growth and proliferation between cells treated with 150 uM of dihydromyricetin and DMSO control solvent in RBE cells. (**C**) Cell migration and invasion abilities were tested by transwell assay. (**D**) Western blot assay was performed to determine the levels of proteins (ZEB1, E-cadherin, Vimentin) associated with EMT. n = 3 independent experiments. Values were given as means ± SEM. **P<0.01.

DMY has shown broad anti-tumor effects in multiple cancers without adverse side effects, therefore, the safety and effect of DMY on cell growth of the RBE cell line was first assessed by the CCK-8 assay. As shown in Figure S1, DMY inhibited the growth of RBE cells in a dose-dependent manner, and its half maximal inhibitory concentration (IC50) was 146.6 μM. Thus, in the following experiments, we used 150 μM of DMY to treat the CCA cell lines. Next, the colony-forming assay was performed and the result demonstrated that DMY suppressed about 70% of cell growth in RBE cells compared to the controls (Figure 1A). The EdU assay showed that DMY significantly inhibited cell proliferation in RBE cells (Figure 1B). Because one of the most important issues in treating patients with CCA is the high metastatic capability of CCA cells, we attempted to investigate DMY's inhibitory effect on migration and the underlying mechanism in CCA in this study. As epithelial-to-mesenchymal transition (EMT) is one of the most essential mechanisms of enhancing cancer cell migration ability in multiple tumors including CCA 5, 6, we performed several functional experiments in the subsequent experiments. As shown in Figure 1C, 150 μM of DMY treatment significantly inhibited cell migration and invasion in both RBE cells and TFK-1 cells as determined by the transwell assay. Moreover, Western blotting showed that DMY treatment significantly altered the expression of EMT-related marker genes, including reduced vimentin and ZEB1 protein expressions and increased E-cadherin expression compared to the control group in both RBE and TFK-1 cells (Figure 1D). Collectively, these data suggest that DMY inhibits cell growth and EMT in cholangiocarcinoma cells.

### The miR-455-3p/ZEB1 axis is involved with EMT in CCA and may be the target of DMY

**Figure 2:**
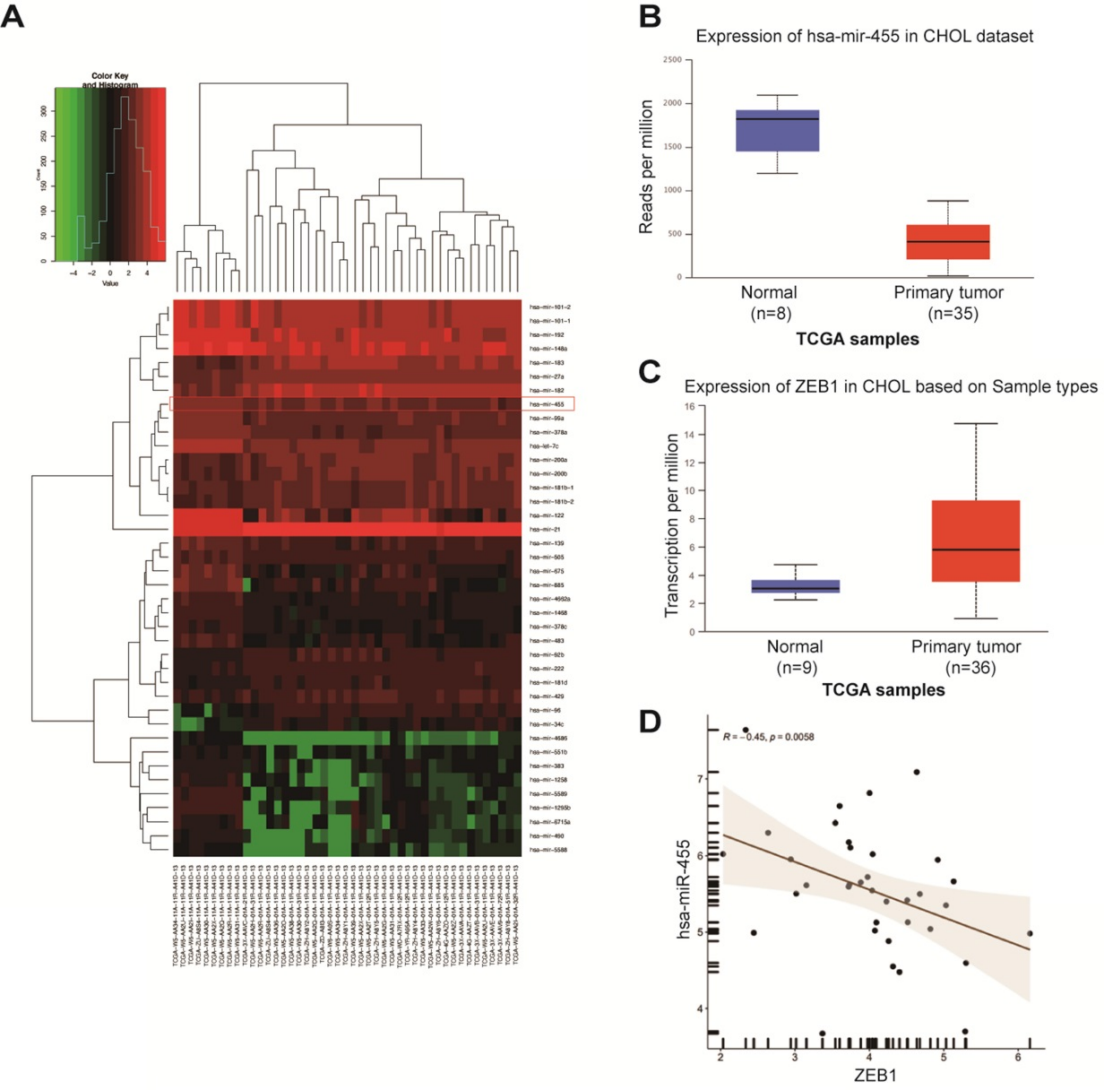
Expression of miR-455 and ZEB1 in human cholangiocarcinoma tissue. (**A**-**C**) TCGA dataset for analyzing the expressions of miRNAs, miR-455 and ZEB1 in human cholangiocarcinoma tissue. (**D**) Expression of ZEB1 was negatively related to the expression of miR-455 as analyzed by the Spearman's correlation analysis.

Accumulating data have proven that miRNAs are potential upstream regulators of EMT in cancer cells, including CCA 12, 13. Therefore, we used TargetScan algorithm (http://www.targrtscan.org/; last accessed March 20, 2021) and predicted that ZEB1 was a potential target of miR-455-3p. MiR-455 is involved in development of diverse malignancies, and a previous study in human keratinocyte (HaCaT) cells demonstrated that the miR-455/ZEB1 axis played an important role in EMT during arsenite-induced malignant transformation 18. Thus, we next investigated whether miR-455/ZEB1 axis-mediated EMT was also involved in CCA and DMY's anti-EMT effects. In order to answer these questions, we first explored the expressions of miR-455 and ZEB1 in human CCA tissues compared to the normal samples by analyzing the TCGA dataset. As shown in Figure 2A, miR-455 expression was significantly decreased in tumor tissue compared to normal tissues in human CCA in a microarray data extracted from TCGA dataset. This observation was further confirmed in a specific data set derived from TCGA webset (Figure 2B). Conversely, the expression of ZEB1 in CCA tissues was remarkably up-regulated (Figure 2C). Moreover, the expression of ZEB1 was negatively related to the expression of miR-455 in CCA tissues (Figure 2D), suggesting that ZEB1 may be a target of miR-455 in CCA.

**Figure 3:**
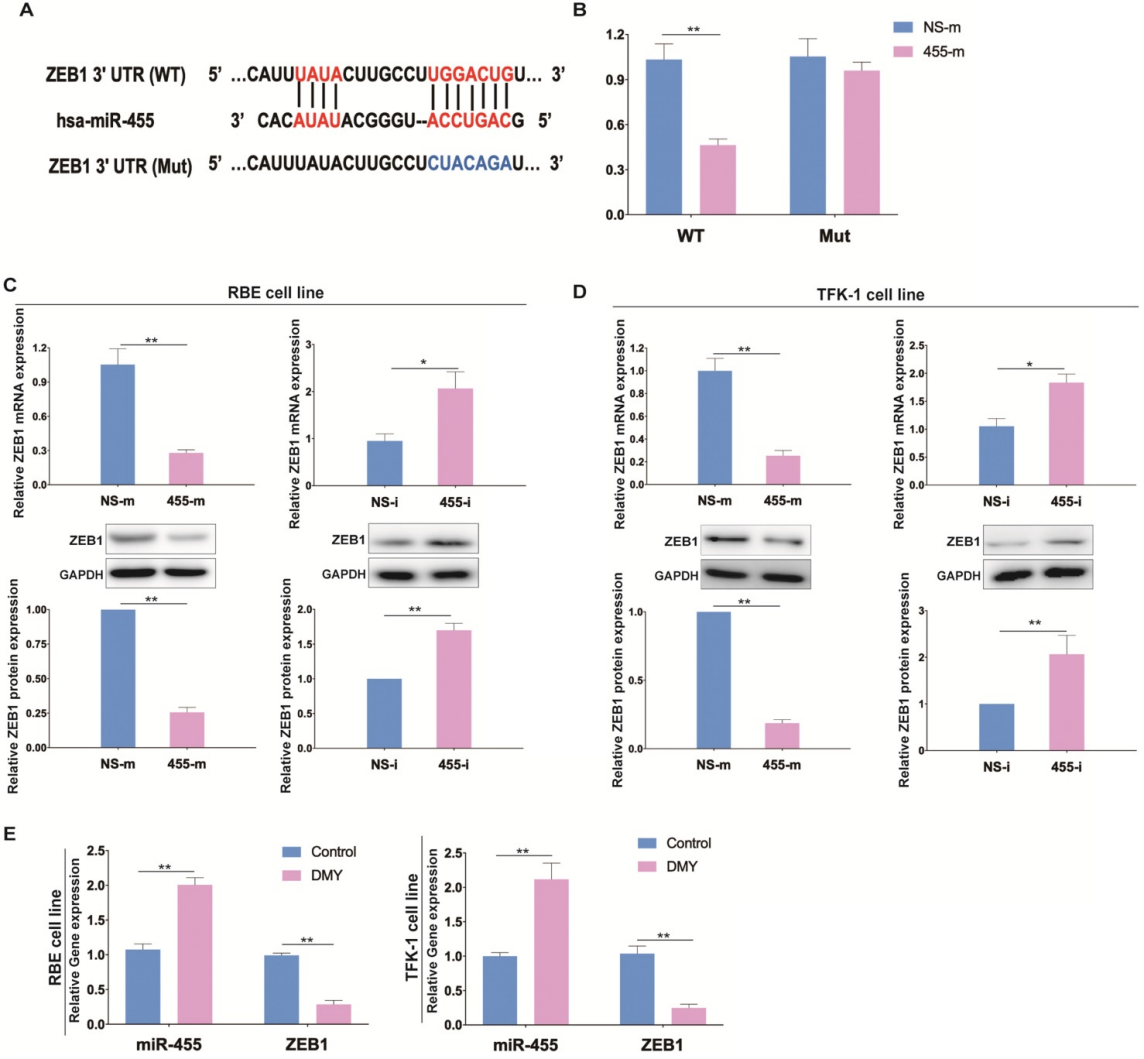
ZEB1 is the target of miR-455 in human cholangiocarcinoma and dihydromyricetin regulates the expressions of miR-455 and ZEB1. (**A**) Schematic graph illustrating the binding sites of miR-455-3p in the 3'-UTR region of ZEB1. (**B**) The relative luciferase activity of wild type or mutant type ZEB1 vector was evaluated by Dual-Luciferase reporter assay system in RBE cells. (**C**-**D**) In RBE cells and TFK-1 cells, the mRNA level and protein level of ZEB1 in cells transfected with NS-i or 455-i (NS-m or 455-m) were respectively measured by real time PCR and western blotting. (**E**) Real time PCR was adopted to measure the RNA level of miR-455 and ZEB1 after exposure to 150 uM of dihydromyricetin or DMSO control solvent in RBE cells and TFK-1 cells. n = 3 independent experiments. Values were given as means ± SEM. * *P* < 0.05 and ** *P* < 0.01.

Next, a luciferase reporter assay was performed to confirm the association between miR-455-3p and ZEB1 in CCA (Figure 3A). As shown in Figure 3B, cells transfected with miR-455-3p mimics (455-m) exhibited a significantly reduced luciferase activity compared to those cells transfected with non-specific mimics (NS-m), indicating that ZEB1 is a direct target of miR-455-3p. Next, we investigated whether miR-455-3p exerted its function through controlling ZEB1 expression in CCA cells. We found that in both RBE and TFK-1 cells which were transfected with 455-m showed significantly reduced ZEB1 expression (Figure 3C). Conversely, down-regulation of miR-455-3p using miR-455-3p inhibitor (455-i) markedly increased the expressions of ZEB1 at both mRNA and protein level (Figure 3D). The efficiencies of 455-m and 455-i were confirmed by qPCR (Figure S2). Moreover, we also tested the expressions of miR-455-3p and mRNA level of ZEB1 after exposure to DMY in both RBE cells and TFK-1 cells as assessed by real time PCR. As shown in Figure 3E, DMY treatment significantly increased the expression of miR-455-3p and decreased the mRNA level of ZEB1 compared to control cells. Taken together, these data demonstrated that ZEB1 is the direct target of miR-455-3p in CCA and the underlying mechanism of DMY mediated-EMT inhibition effects in CCA may be through regulating the miR-455-3p/ZEB1 axis.

### Down-regulation of miR-455-3p abolishes DMY's inhibitory effect on cell proliferation and EMT in CCA cells

**Figure 4:**
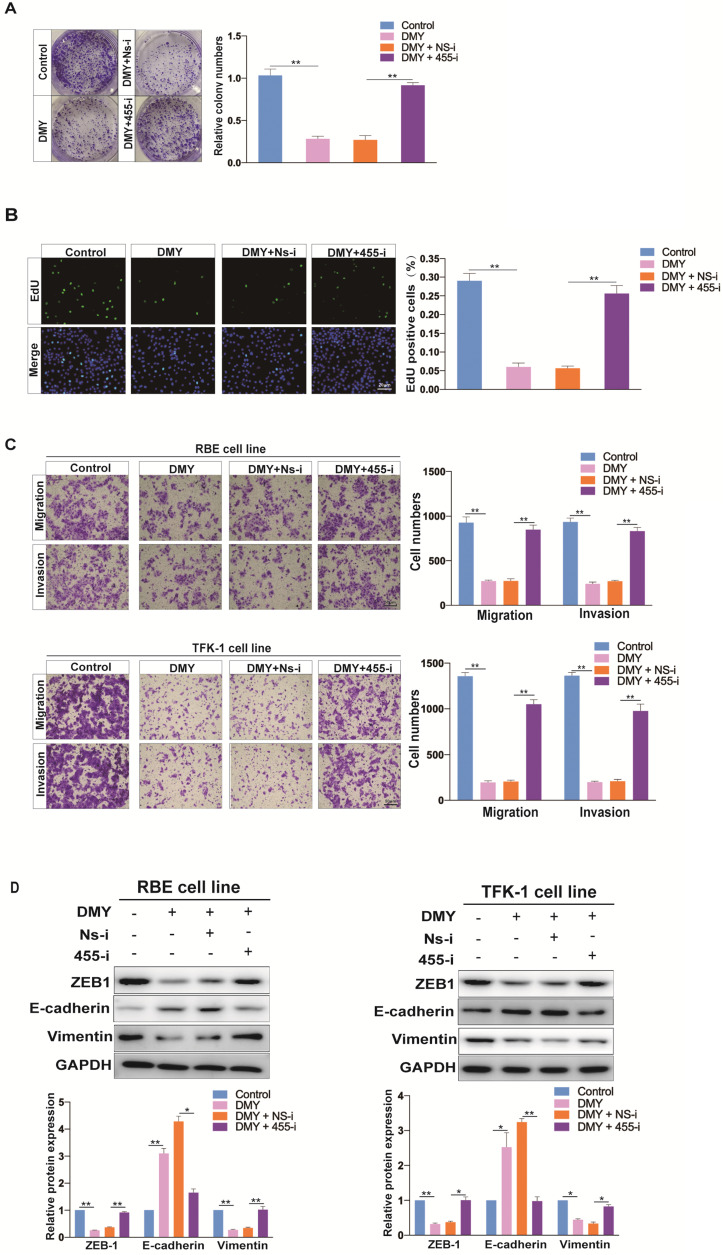
Downregulation of miR-455 abolishes dihydromyricetin' s inhibitory effect on cell proliferation and EMT in CCA cells. RBE cells and TFK-1 cells were transfected with miR-455-3p inhibitor (455-i) or non-specific inhibitor (NS-i). (**A**) Colony formation assay in RBE cells. (**B**) Edu assay in RBE cells. (**C**-**D**) Cell invasion and migration evaluated by transwell assay respectively in RBE cells and TFK-1 cells. (**E**) EMT related protein levels (ZEB1, E-cadherin, Vimentin) evaluated by Western blot assay in RBE cells and TFK-1 cells. n = 3 independent experiments. Values were given as means ± SEM. * P < 0.05 and ** *P* < 0.01.

In order to confirm that DMY inhibited EMT in CCA was through regulating miR-455-3p, we tested if inhibition of miR-455-3p expression using 455-i would abolish DMY's anti-tumor effects in CCA cell lines. Figure 4A and 4B showed that down-regulation of miR-455-3p remarkably abolished the inhibitory effect of DMY on cell growth and proliferation in REB cells as assessed by the colony formation assay and EdU assay. In addition, the transwell assay in both RBE cells and TFK-1 cells revealed that inhibition of miR-455 abrogated DMY-mediated suppressing effects on cell migration and invasion (Figure 4C). Consistently, compared to RBE or TFK-1 cells transfected with NS-i and treatment with DMY, Western blot showed that down-regulation of miR-455-3p increased the expressions of vimentin and ZEB1 in those DMY-treated cells, while decreased the expression of E-cadherin (Figure 4D). Taken together, these data demonstrated that down-regulation of miR-455-3p abolished the inhibitory effects of DMY on cell proliferation and EMT in CCA cells, indicating that the mechanism of DMY's inhibitory effect was through regulating miR-455-3p.

### Inhibition of miR-455-3p abrogates DMY-mediated inhibitory effects on tumor growth and EMT in human CCA cell-engrafted nude mice

**Figure 5:**
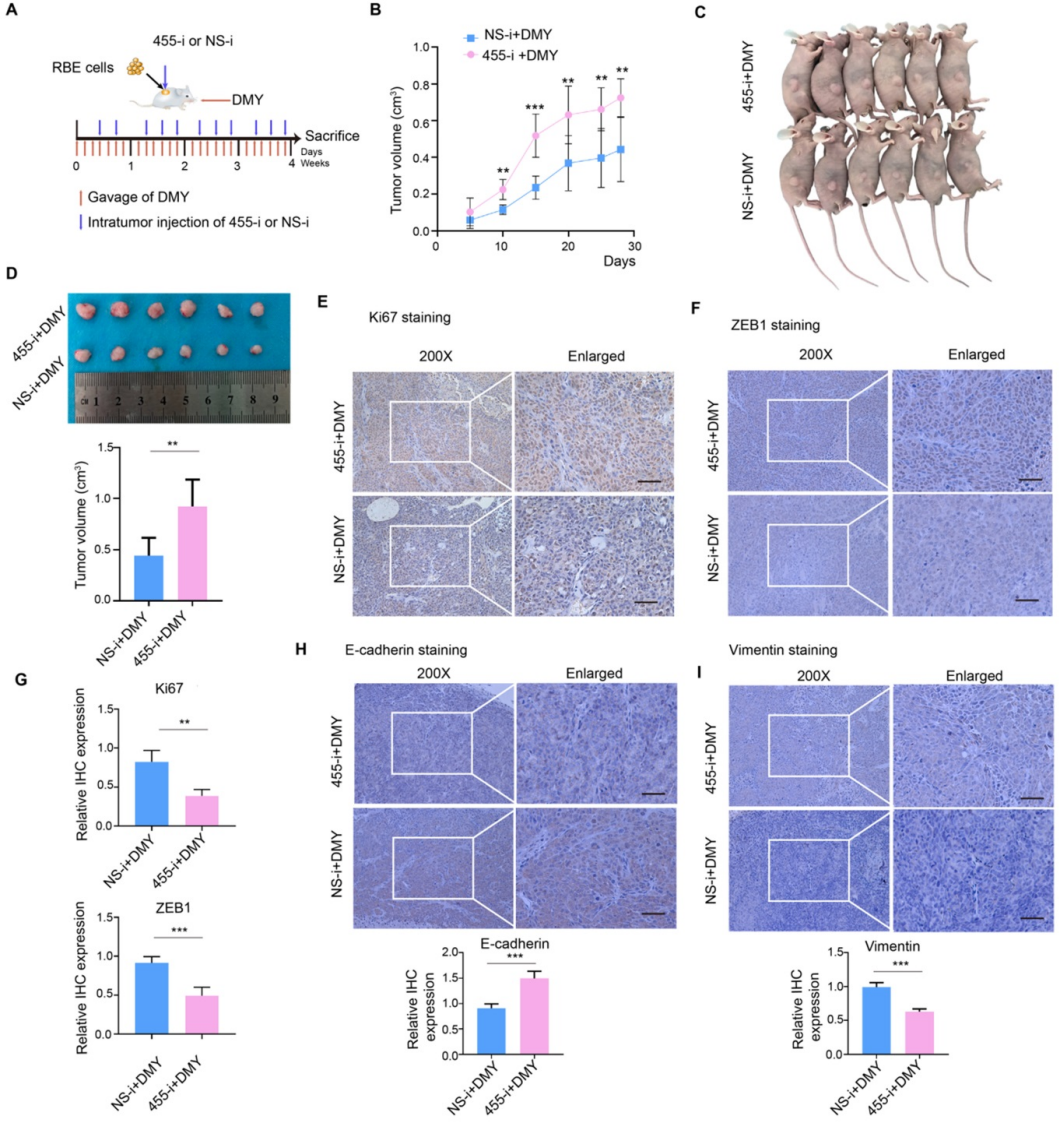
Inhibition of miR-455-3p abrogates dihydromyricetin-mediated inhibitory effects on tumor growth and EMT in human cholangiocarcinoma cell-engrafted nude mice. (**A**) Schematic graph illustrating the protocol of intratumoral injection with lipofectamine 2000 encapsulated miR-455-3p inhibitor (455-i) or non-specific inhibitor (NS-i) (5 nM/mouse) and gavaged with 500 mg/kg/day dihydromyricetin for four weeks. (**B**) Tumor growth curve over time of human cholangiocarcinoma cell-engrafted nude mice treated with dihydromyricetin and NS-i or 455-i, respectively. (**C**-**D**) Tumor image and quantification of tumor volume after mice treated with dihydromyricetin and NS-i or 455-i on day 29, respectively. (**E**, **F, H, I**) Immunohistochemistry staining and quantification of Ki67 and ZEB1, E-cadherin and Vimentin in xenograft tissues after mice treated with dihydromyricetin and NS-i or 455-i, respectively.** (G)** Histograms of the relative IHC expression of Ki67 and ZEB1. Scar bar 100 μM, insert 50 μM. *n* = 6 mice per group. Values are given as means ± SEM. ** *P* < 0.01 and *** *P* <0.001.

Subsequently, we further investigated the role of miR-455-3p in DMY-mediated EMT inhibitory effect in human CCA cell-engrafted nude mice. RBE cells (5 × 10^6^) were subcutaneously injected into four-week old Balb/c male nude mice. Three days after injection, the mice were randomly divided into two groups and gavaged with 500 mg/kg/day DMY for four weeks. Figure 5A describes the protocol of intratumoral injection with lipofectamine 2000 encapsulated 455-i or NS-i (5 nM/mouse). On day 29, the mice were sacrificed and tumors were harvested for further analyses. As shown in Figure 5B, the tumors grew much faster in mice injected with 455-i compared to the paired mice injected with NS-i. Figure 5C and 5D showed that the tumor volumes in the group injected with 455-i were significantly larger those injected with NS-i. Moreover, the immunohistochemistry staining and quantification of xenograft tissues showed that down-regulation of miR-455-3p *in vivo* significantly increased the protein expressions of ki67, vimentin and ZEB1, while decreased the protein level of E-cadherin (Figure 5E-H). Collectively, these data demonstrated that inhibition of miR-455-3p abrogated DMY-mediated inhibitory effects on tumor growth and EMT in human CCA cell-engrafted nude mice, further supporting the mechanism of DMY's inhibitory effect on tumor growth and EMT in human CCA was through regulating miR-455-3p.

### DMY inhibits the PI3K/AKT signaling pathway through regulating miR-455-3p in CCA cells

**Figure 6:**
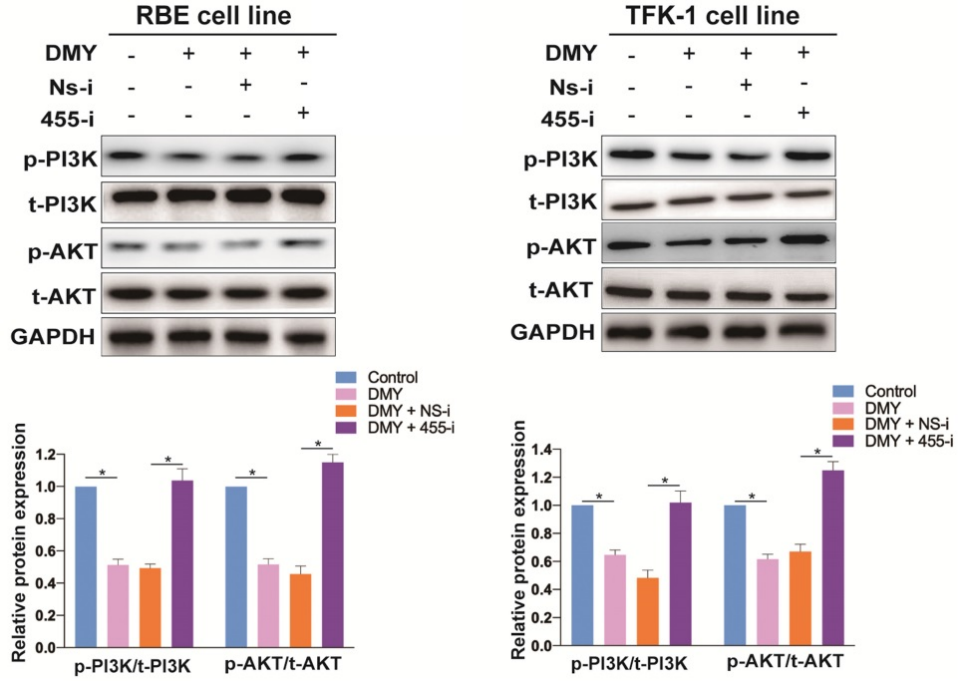
Dihydromyricetin inhibits the PI3K/AKT signaling pathway through regulating miR-455-3p in CCA cells. RBE cells and TFK-1 cells were transfected with miR-455-3p inhibitor (455-i) or non-specific inhibitor (NS-i). Protein expressions were evaluated by Western blot. n = 3 independent experiments. Values were given as means ± SEM. * P < 0.05.

Previous studies have demonstrated that the ZEB1/PI3K/AKT signaling pathway plays an important role in cell growth and invasion in several cancers 19, 20. Thus, we were interested to investigate if DMY could inhibit the ZEB1 downstream PI3K/AKT signaling pathway through regulating miR-455-3p in CCA. As shown in Figure 6, compared to the control cells, DMY treatment significantly suppressed the expression ratio of the phosphorylated PI3K (p-PI3K) to total PI3K (t-PI3K), and the expression ratio of the phosphorylated AKT (p-AKT) to total AKT (t-AKT) was also remarkably attenuated following DMY treatment in both RBE cells and TFK-1 cells as determined by Western blot. However, compared to cells transfected with NS-i, cells transfected with 455-i remarkably enhanced the expression ratios of both p-PI3K to t-PI3K and p-AKT to t-AKT, indicating that down-regulation of miR-455-3p reversed the inhibitory effect of DMY on the PI3K/AKT signaling pathway. In brief, our study confirmed that DMY inhibited EMT in CCA through regulation of the miR-455/ PI3K/AKT pathway.

## Discussion

With advance in the understanding of molecular mechanisms of tumorigenesis and tumor progression, the discovery and development of bioactive compounds from natural plants for the treatment of different cancers has aroused great interest of scientists and healthcare workers 21, 22. DMY, the most abundant natural flavonoid and active compound in Ampelopsis grossedentata W.T. Wang (Vitaceae), has been used as herbal tea and traditional Chinese medicines for over hundreds of years in China. In the past decades, DMY has been widely investigated and shows broad anti-tumor effects. For instance, DMY enhanced the anti-tumor effect of irinotecan in colon cancer in mouse models 8. Wang et al showed that DMY reduced cell migration and invasion and induced cell apoptosis through downregulation of the golgi reassembly and stacking protein 65 (GRASP65) in human ovarian cancer cells 9. In our previous study, we identified that DMY significantly inhibited cell proliferation, migration, invasion and promoted apoptosis in human CCA cell lines via regulating the miR-21/ PTEN/ Akt pathway 11. In this study, we not only confirmed the anti-tumor effect of DMY in a human CCA cell-engrafted nude mice model, but also showed that DMY inhibited EMT through targeting miR-455/ZEB1 axis, an important mechanism associated with tumor initiation and progression in various cancer types, in human CCA both *in vitro* and *in vivo* experiments. This study provides novel evidence that the anti-cancer properties of DMY in CCA through inhibiting EMT, indicating that DMY may be an alternative option for the future treatment of CCA.

The characteristics of high aggressiveness and chemoresistance of CCA contribute to the poor prognosis. Therefore, it is very important to develop new treatment agents based on the understanding of pathogenic molecular mechanisms of CCA. EMT is a biological program involved in the process of organ development and wound healing. However, when EMT is hijacked by cancer cells, this process is often associated with increased tissue invasiveness, cancer stem cell characteristics and resistance to therapeutic regimens 23. Studies have shown that the induction of EMT contributed to the progression of CCA 24, and CCA cells with EMT-like features could induce immunosuppression 25. A review has extensively overviewed different EMT markers investigated in CCA 26. When EMT occurs in cancer, epithelial cancer cells are detached from each other and the basement membrane, then epithelial cancer cells gradually obtain mesenchymal traits with invasive properties after a new transcriptional program is activated, resulting in greater potential of metastatic colonization and therapy resistance 27. Initially believed as a binary process, the recent researches have demonstrated that EMT occurs in a dynamic process which confers to intermediate cellular states with both epithelial and mesenchymal features, leading to cell heterogeneity, tumorigenesis and cancer progression 27, 28.

EMT is regulated by transcription factors (EMT-TFs), including zinc finger proteins (e.g., SNAI1 and SNAI2), basic helix-loop-helix transcription factors (e.g., the TWIST family and E47) and zinc finger and homeodomain proteins (ZEB1 and ZEB2) 29. In this study, we performed the relationship analyses between the expressions of the EMT-TF and miR-455 in human CCA tissues by using the TCGA dataset. As shown in Figure S3, the expression of neither SNAIL nor TWIST was statistically related to the level of miR-455, while the expression of ZEB1 was negatively associated with the expression of miR-455. Therefore, we focused on ZEB1. The Kaplan-Meier analysis (Figure S3) did not demonstrate positive correlation between miR-455-3P (or ZEB1) and CCA patient survival time, probably these was due to small sample sizes. Larger clinical samples are required to verify the results. ZEB1 is considered as a driver of EMT and cancer progression. For example, ZEB1 could down-regulate the expression of E-cadherin, a major cell-cell adhesion molecule, and up-regulate the expression of vimentin, a key protein for EMT and cancer metastasis 29, 30. In our study, the functional experiments of the transwell assays demonstrated that DMY treatment suppressed the migration and invasion of CCA cell lines, which in line with EMT inhibition, indicated by decreased ZEB1 and its downstream protein of vimentin expression and upregulated E-cadherin expression in CCA cells treatment with DMY.

MiRNAs act as oncogenes or tumor suppressor genes in controlling tumor initiation, progression and metastasis. The role of miR-455 has been investigated in several cancers. For instance, in human breast cancer cells, miR-455 inhibited cell proliferation through targeting CDK14 31. MiR-455 suppressed cell growth and invasion through targeting SMAD2 in pancreatic cancer cells. Moreover, this study found that low expression of miR-455 and high level of SMAD2 were associated with poor patient survival 22. In non-small cell lung cancer, miR-455 also exhibited anti-tumor property. MiR-455 inhibited the proliferation, migration, and invasion of non-small cell lung cancer cell lines by targeting ZEB1. Overexpression of ZEB1 reversed the inhibitory effect of miR-455 16. Similarly, a recent study also showed that miR-455 could inhibit EMT through targeting ZEB1 in arsenite-induced malignant transformation of human keratinocytes 18. However, the role of miR-455 in CCA is still not clear. Our study found that miR-455 was down-regulated in CCA tissues compared to normal tissues, and overexpression of miR-455-3p inhibited ZEB1 expression in human CCA. Importantly, our study demonstrated that DMY increased the expression of miR-455-3p, while decreased the expression of ZEB1. In support, down-regulation of miR-455-3p expression abolished the inhibitory effects of DMY on cell proliferation and EMT *in vitro* and *in vivo*. Together with our previous study 11, our data indicated that the anti-tumor effects of DMY's on CCA is through a synthetic mechanism associated with multiple targets, such as miR-455 and miR-21.

The PI3K/AKT signaling pathway contributes importantly to tumor proliferation and metastasis in multiple human malignancies, including CCA 32, 33. Anti-tumor agents such as anlotinib inhibits tumor progression via targeting the PI3K/AKT pathway in intrahepatic cholangiocarcinoma 34. AGO1 enhances proliferation and invasion through modulating EMT-associated related TGF-β-PI3K-AKT signaling pathways in cholangiocarcinoma 5. The ZEB1/PI3K/AKT signaling pathway also plays an important role in tumor cell growth and invasion 19, 20. For instance, in non-small cell lung cancer cells, downregulation of Rhomboid domain containing 1 (RHBDD1) expressions inhibits cell growth and invasion through reducing the ZEB1/PI3K/AKT signaling pathway activation 19. In the current study, we showed that DMY treatment inhibited the expression of ZEB1, p-PI3K and p-AKT in both RBE cells and TFK-1 cells, which in line with increased miR-455 expression. In agreement with this observation, inhibition of miR-455 abrogated the anti-tumor effects of DMY on CCA, as well as ZEB1, p-PI3K and p-AKT expression, indicating that DMY inhibits EMT in CCA through targeting the miR-455/PI3K/AKT pathway (Figure S4).

## Conclusion

In conclusion, our study demonstrated that DMY suppressed cell proliferation, tumor growth and EMT through regulating miR-455-3p in CCA. We believe this study will help us to better understand the effects of DMY in human CCA and these findings suggest that DMY may be explored as a therapeutic option for the treatment of CCA.

## Supplementary Material

Supplementary figures.

## Abbreviations

CCA: cholangiocarcinoma
CCK-8: Cell Counting Kit-8
DMY: Dihydromyricetin
ECL: electrochemiluminescence
EMT: epithelial-mesenchymal transition
IC50: half maximal inhibitory concentration
MET: mesenchymal-epithelial transition
NS-i: non-specific inhibitors
NS-m: non-specific mimics
455-m: miR-455-3p mimics
455-i: miR-455-3p inhibitors
PVDF: polyvinylidene fluoride
SDS-PAGE: sodium dodecyl sulfate - polyacrylamide gel electrophoresis
TF: transcription factor
