# Supplementary material for: Dihydromyricetin Inhibits Tumor Growth and Epithelial-Mesenchymal Transition through regulating miR-455-3p in Cholangiocarcinoma
Source: J Cancer. 2021 Aug 22;12(20):6058–70. doi: 10.7150/jca.61311 (PMC8425191; doi:10.7150/jca.61311)
Supplement: Supplementary file 1 — Supplementary figures. [file jcav12p6058s1.pdf]

Supplementary Figures

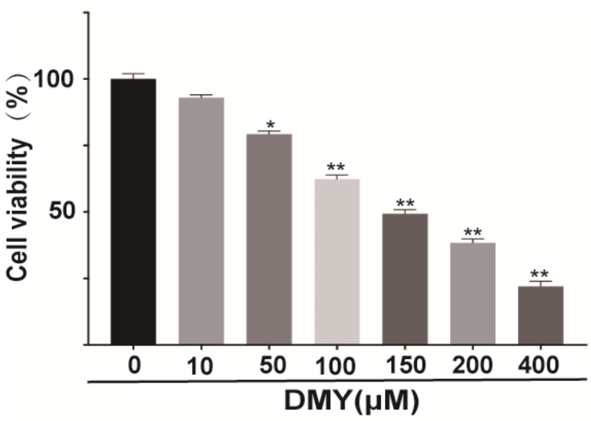

Figure S1 Dihydromyricetin inhibits RBE cells' viability in a dose-dependent manner. The cell viability after exposure to different concentrations of dihydromyricetin for 24 hours in RBE cells was determined by CCK-8 assay. n = 3 independent experiments. Values were given as means ± SEM. * $P < 0.05$, ** $P < 0.01$.

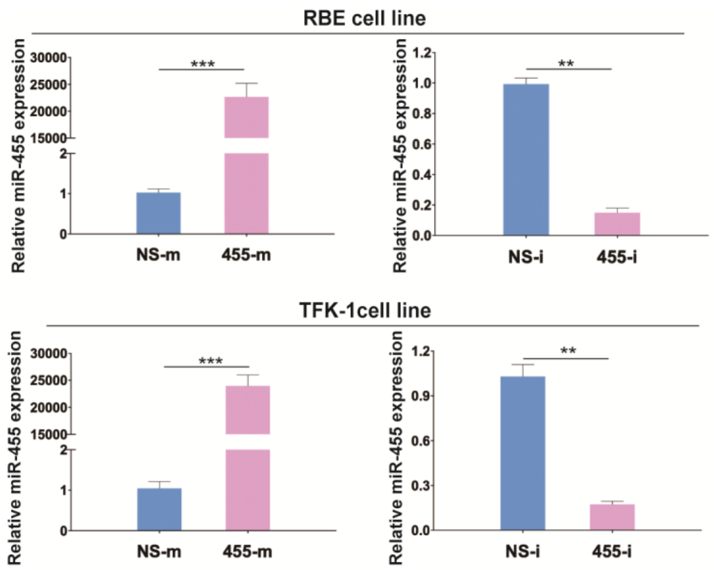

Figure S2 The transfection efficiency of miR-455-3p mimics and inhibitors. RBE cells and TFK-1 cells were transfected with miR-455-3p mimics (455-m) or non-specific mimics (NS-m), or with miR-455-3p inhibitors (455-i) or non-specific inhibitors (NS-i). The mRNA level of miR-455 was evaluated by real time PCR. n = 3 independent experiments. Values were given as means ± SEM. * $P < 0.05$, ** $P < 0.01$.

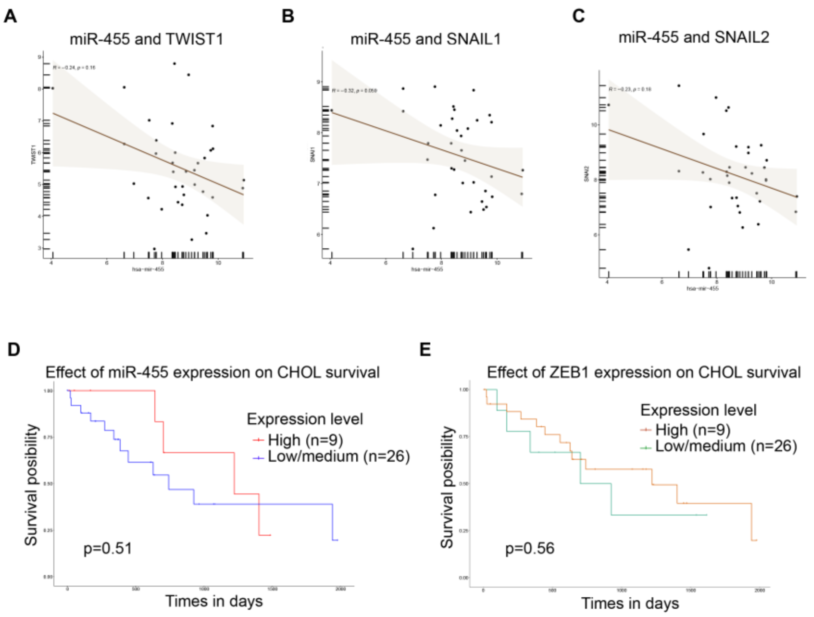

Figure S3 Correlation analyses between miR-455 and the EMT- transcription factors and the relationship between miR-455 (or ZEB1) and patient survival time in human CCA by using the TCGA dataset.

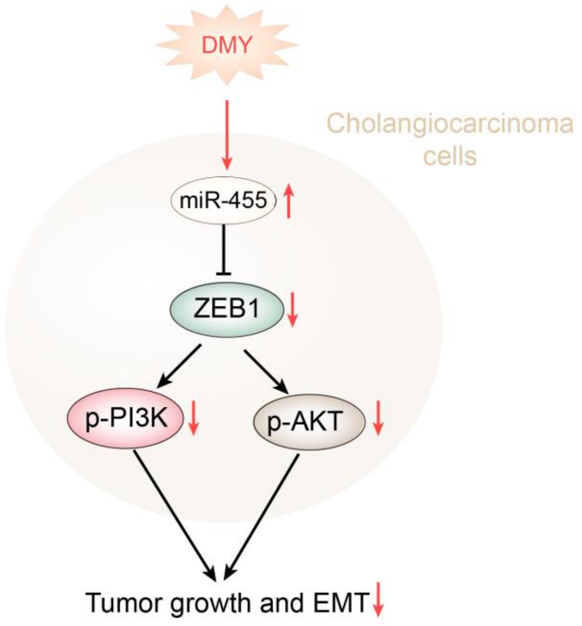

Figure S4 Mechanism diagram of DMY's inhibitory effect on tumor growth and EMT in CCA.
